# Thin zeolite laminates for rapid and energy-efficient carbon capture

**DOI:** 10.1038/s41598-017-10518-4

**Published:** 2017-09-08

**Authors:** Farid Akhtar, Steven Ogunwumi, Lennart Bergström

**Affiliations:** 1 Division of Materials Science, Luleå University of Technology, 97187 Luleå, Sweden; 2 Department of Materials and Environmental Chemistry, Stockholm University, Stockholm, 10691 Sweden; 3 Crystalline Materials Research, Corning Incorporated, Corning, New York USA

## Abstract

Thin, binder-less zeolite NaX laminates, with thicknesses ranging between 310 to 750 μm and widths exceeding 50 mm and biaxial tensile strength in excess of 3 MPa, were produced by pulsed current processing. The NaX laminates displayed a high CO_2_ adsorption capacity and high binary CO_2_-over-N_2_ and CO_2_-over-CH_4_ selectivity, suitable for CO_2_ capture from flue gas and upgrading of raw biogas. The thin laminates displayed a rapid CO_2_ uptake; NaX laminates with a thickness of 310 μm were saturated to 40% of their CO_2_ capacity within 24 seconds. The structured laminates of 310 μm thickness and 50 mm thickness would offer low pressure drop and efficient carbon capture performance in a laminate-based swing adsorption technology.

## Introduction

Zeolites, aluminophophates and carbon molecular sieves are commonly used adsorbents for gas separation, drying and gas purification^1–4^. Typically, the adsorbents are packed into vessels or columns and subjected to a pressure/vacuum swing adsorption process to selectively adsorb and desorb gas species^5^. Important requirements for adsorbents to be used in e.g. carbon capture from large power-plants, and low cost, small-scale solutions for biogas upgrading, include: low pressure drop, high uptake, high selectivity and short cycle time. Adsorbent beds consisting of packed granules or beads are simple to manufacture but suffer from large pressure drop, low volumetric efficiency and slow mass transfer coefficients. In addition, the pelletized or granulated adsorbents experience abrasion and surface wear during pressure cycling and disintegrate into fine dust particles which results in increased down times for swing adsorption equipment. Structured adsorbents in the form of monoliths, foams, honeycombs and laminates have the potential to overcome these problems^4, 6^. Indeed, recent work has suggested that laminated adsorbents could significantly improve the efficiency through a combination of low pressure drop, rapid mass and heat transfer, high volumetric efficiency and extended durability that is needed for volume efficient and rapid swing adsorption technologies^7–9^.

Laminated adsorbents have been produced using a non-adsorbing support e.g. metal foil, woven wire mesh, woven glass fiber mesh etc^10, 11^. Non-adsorbing binders, e.g. clays, are often added to improve the mechanical stability^4, 12, 13^. However, inert, non-adsorbing additives dilute the active components and thus result in a reduced volume efficiency. Hence, the development of new processes to produce self-standing laminated adsorbents with close dimensional tolerances and high mechanical stability without support material or addition of binders is of pivotal importance for cost- and volume-efficient gas separation processes with large flows, especially CO_2_ capture from power-plant flue-gas and biogas upgrading.

Binder-less processing routes of zeolites include hydrothermal transformation of inorganic binders, clay and silica, into zeolitic materials^14–17^ and pulsed current processing (PCP) to directly consolidate microporous powders^4^. We have previously shown that rapid heating of a powder body subjected to a compressive stress can generate binderless adsorbents^18–20^ with a significant mechanical strength from different types of porous powders, including zeolites and aluminophosphates.

In this study, we demonstrate that mechanically stable and thin self-standing laminates of NaX zeolite can be produced by a tailored pulsed current processing (PCP) method from NaX zeolite powder without addition of binders. We show that the laminate thickness can be easily controlled. Maximum PCP temperature and consolidation pressure were optimized to ensure a high mechanical strength and minimal loss of surface area. The PCP-produced laminates with thicknesses between 310 and 750 micrometers display hierarchical porous structure, rapid CO_2_ adsorption kinetics and a high mechanical stability. The thickness dependence of the CO_2_ adsorption kinetics of the laminates will be discussed.

## Results and Discussions

**Figure 1:**
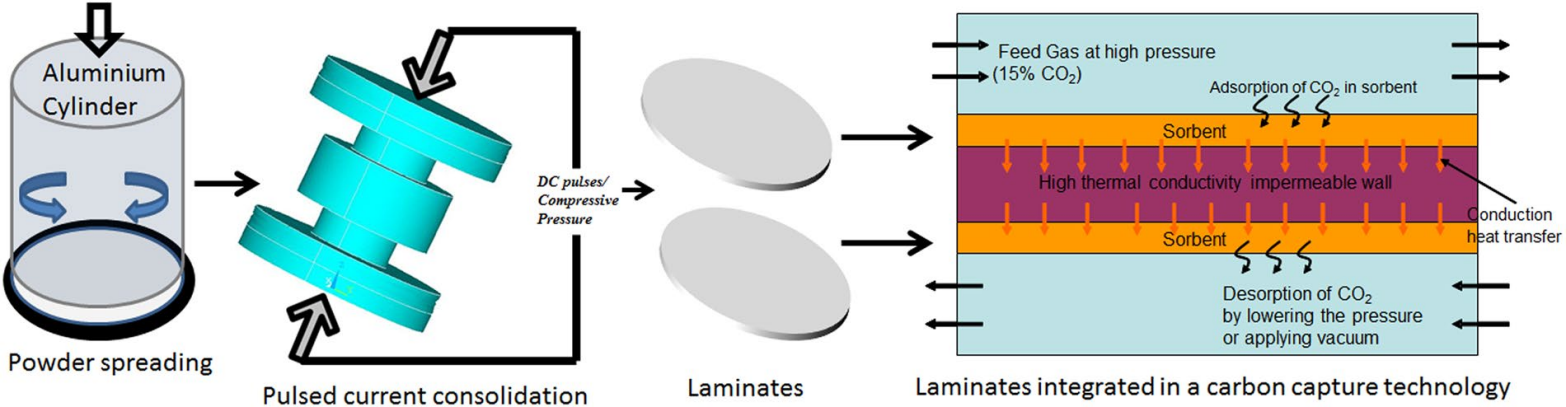
Schematic illustration of processing steps; powder spreading on graphite paper, pulsed current processing and the consolidated laminates. Also shown in a schematic of the concept of the cyclic application of laminates where adsorption takes place in the sorbent (laminate) top layer due to CO_2_ partial pressure difference between the feed gas (higher partial pressure) and the sorbent. The top layer is in direct thermal communication with the bottom layer which drives desorption in the bottom layer. Moreover, lowering of pressure or evacuation could be utilized for desorption cycle. In effect, the energy released during the exothermic adsorption process in the top layer is used by the endothermic desorption process in the bottom layer.

**Figure 2:**
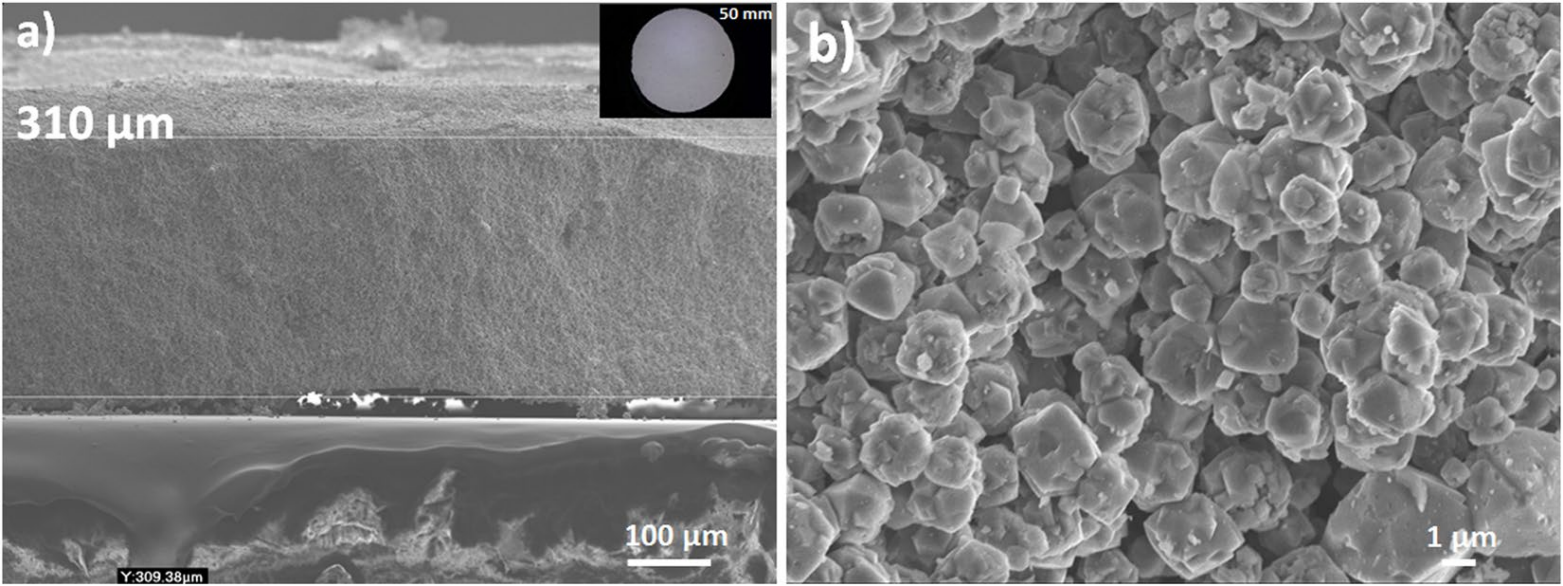
(**a**) SEM micrograph of NaX laminate; (**b**) NaX particles in the laminate. The inset in (**a**) shows the laminate of 50 mm in diameter.

Zeolite NaX laminates of thicknesses between 310 to 750 μm were produced by pulsed current processing (PCP). Thin laminates were obtained by spreading a specific amount of NaX powder homogeneously onto a graphite paper that was placed into the PCP mold. The uniform distribution and height of the powder on the graphite paper was assured by depositing the powder while the graphite paper was rotated and the loose powder layer was consolidated by prepressing at 50 MPa. The combination of pre-deposition of a thin and homogeneous powder layer and optimization of the PCP processing conditions produced self-standing binder-free NaX laminates with a dimensional variation of 15–20 µm (Table 1). The fabrication steps of the laminates are summarized in Fig. 1 together with a conceptual illustration on how structured laminates can be used in rapid swing adsorption processes where it is essential to minimize the pressure drop and heat accumulation^4, 6, 7^. Laminates with diameters up to 50 mm could be fabricated by PCP of pre-deposited the homogeneous powder layers (Fig. 2). The Scanning electron micrographs (SEM) of a representative zeolite NaX laminate illustrate uniform thickness of 310 µm (Fig. 2a). The high resolution electron micrograph of NaX laminate (Fig. 2b) shows that the NaX crystals stay intact during PCP thermal treatment(see Supplementary Information, section S1). The large and thin NaX laminates are suitable for constructing adsorption based devices consisting of stacks of laminates placed with a small gap that serves as flow channels for gas transport (Fig. 1). Keefer *et al*. suggested that a laminate device with flow channel length of 100–200 mm and 50–75 µm channel width could operate at high cyclic frequencies^21^.

**Table 1:** Textural properties, average micropore size, total macropore area, porosity and biaxial strength of 13X laminate.

Specimen	Laminate Thickness (μm)	^a^BET surface area (m^2^/g)	^a^t-Plot Micropore Area (m^2^/g)	^a^External Surface Area (m^2^/g)	^b^Average Macropore diameter (µm)	^b^Porosity (%)	Biaxial Strength (MPa)
13X Powder	—	742	711	31	—	—	—
Laminate PCP at 510 °C	310 ± 15	695 ± 15	669	37	0.55	40	4.0 ± 0.4
Laminate PCP at 510 °C	600 ± 20	682 ± 10	642	40	0.61	39	3.2 ± 0.2
Laminate PCP at 510 °C	750 ± 20	687 ± 10	651	36	0.56	41	4.5 ± 0.2

^a^Determined from N_2_ adsorption at 77 K; ^b^determined from mercury instrusion porosimetry.

The biaxial tensile strength of PCP-consolidated binderless NaX laminates is over 3 MPa (Table 1). The biaxial tensile strength of the laminates is comparable to zeolite monoliths produced by PCP and by conventional processing methods incorporating significant amounts of inorganic binders^9, 18, 19, 22^. Biaxial strengths of 3 MPa are sufficient for low pressure swing adsorption processes where the pressure is altered between 1 bar and 6 bar^23, 24^. Moreover, in temperature swing based technologies, the binderless and homogeneous NaX laminates are expected to be superior to conventional laminates prepared with clay binders where the difference in thermal expansion between the clay and the zeolite could result in thermal cracking and degradation^25, 26^.

Mercury intrusion porosimetry data (Table 1) shows that the laminates display a hierarchical porosity with micropores defined by the structure and intrinsic porosity of the zeolite crystals, and macropores that are intraparticle voids between the NaX crystals. Previous work has shown that a hierarchical porous structure is essential for rapid gas adsorption and desorption^5, 18^. Optimization of the pressure and temperature during the PCP treatment retained the intraparticle voids in the PCP processed laminates due to small (local) densification at the particle contacts. The total porosity of 40% corresponds to the particle packing density of such non-spherical particles. The NaX laminates show a relatively small reduction of the BET surface area compared to the NaX powder, which suggests that the PCP-treatment only results in the formation of an amorphous phase locally at the contact points of adjacent NaX particles^4, 18, 20^. The NaX laminates of different thicknesses show comparable BET surface area suggesting that the pulsed current processing homogeneously consolidated the NaX powder into structured laminates of varying thicknesses.

**Figure 3:**
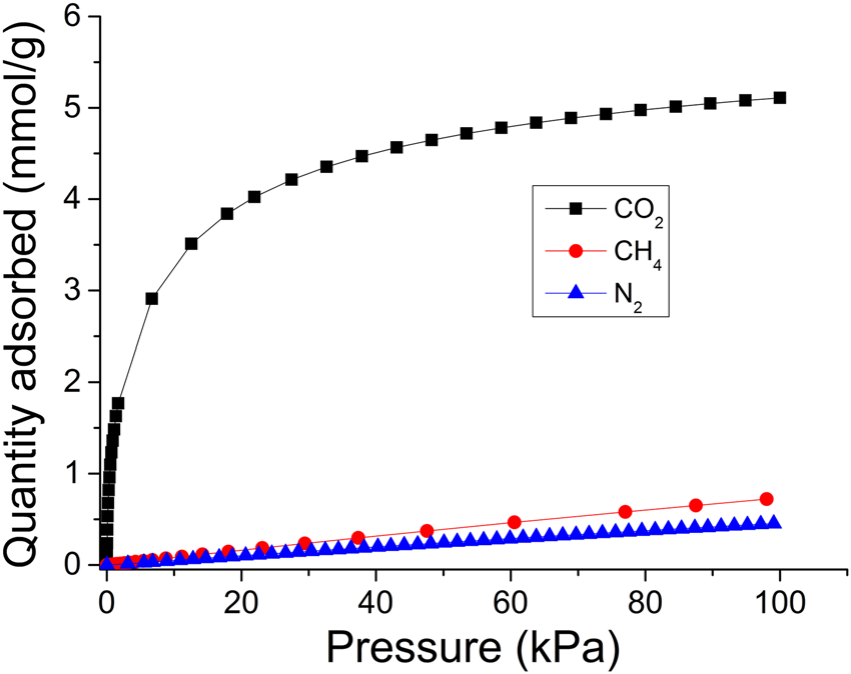
CO_2_, CH_4_ and N_2_ adsorption isotherms of 310 µm NaX laminate at 25 °C.

The adsorption isotherms (Fig. 3) recorded on a 310 μm thick NaX laminate show that CO_2_ adsorption capacity is high (5.0 mmol/g at 101 kPa at 25 °C) while the adsorption capacity of N_2_ and CH_4_ is significantly lower. We find that the PCP treatment does not influence significantly the high CO_2_ adsorption of zeolite NaX (see Supplementary Information, section S2), which is related to the strong interaction of the surfaces of the ionic aluminosilicate micropore cages with CO_2_^18, 27^.

Binary CO_2_-over-N_2_ selectivity and CO_2_-over-CH_4_ selectivity are important parameters for decarbonisation of flue gas and raw biogas. We estimate CO_2_-over-N_2_ and CO_2_-over-CH_4_ thermodynamic selectivities using ideal adsorbed solution (IAS) theory^28^ as follows

1 $${\alpha }_{C{O}_{2}/{N}_{2}}=\frac{{x}_{C{O}_{2}}.{y}_{{N}_{2}}}{{x}_{{N}_{2}}.{y}_{C{O}_{2}}}$$

2 $${\alpha }_{C{O}_{2}/C{H}_{4}}=\frac{{x}_{C{O}_{2}}.{y}_{C{H}_{4}}}{{x}_{C{H}_{4}}.{y}_{C{O}_{2}}}$$

Where ${x}_{C{O}_{2}}$, ${x}_{C{H}_{4}}$ and ${x}_{{N}_{2}}$ are mole fraction of CO_2_, CH_4_ and N_2_ in the adsorbed state and ${y}_{C{O}_{2}}$, ${y}_{C{H}_{4}}$ and ${y}_{{N}_{2}}$ are mole fraction of CO_2_, CH_4_ and N_2_ in the gas phase. Table 2 shows that the binary CO_2_-over-N_2_ selectivity is 290 and CO_2_-over-CH_4_ is 96. These high binary selectivities meet the criteria of efficient CO_2_ capture from power plant flue-gas and biogas upgrading^27, 29^.

**Table 2:** CO_2_, CH_4_ and N_2_ adsorption parameters, Henry’s law constant and IAST CO_2_-over-N_2_ and CO_2_-over-CH_4_ selectivities of 310 μm laminates prepared by pulsed current processing.

Adsorbate	[a]q_m_ (mmol/g)	[a]b (1/kPa)	K_H_ (q_m_ × b)	[b]Binary CO_2_/N_2_ Selectivity	[b]Binary CO_2_/CH_4_ Selectivity
CO_2_	4.98	0.252	1.254	290	96
CH_4_	6.72	0.00123	0.00827	—	—
N_2_	4.24	0.00121	0.00514	—	—

[a] Acquired from CO_2_, CH_4_ and N_2_ adsorption isotherm, respectively, at 298 K by implementing Langmuir model to data. [b] Calculated IAST at 100 kPa in 15 mol% CO_2_ and 85 mol% N_2_ and 50 mol% CO_2_ and 50 mol% CH_4_ binary mixtures at 100 kPa and 25 °C.

**Figure 4:**
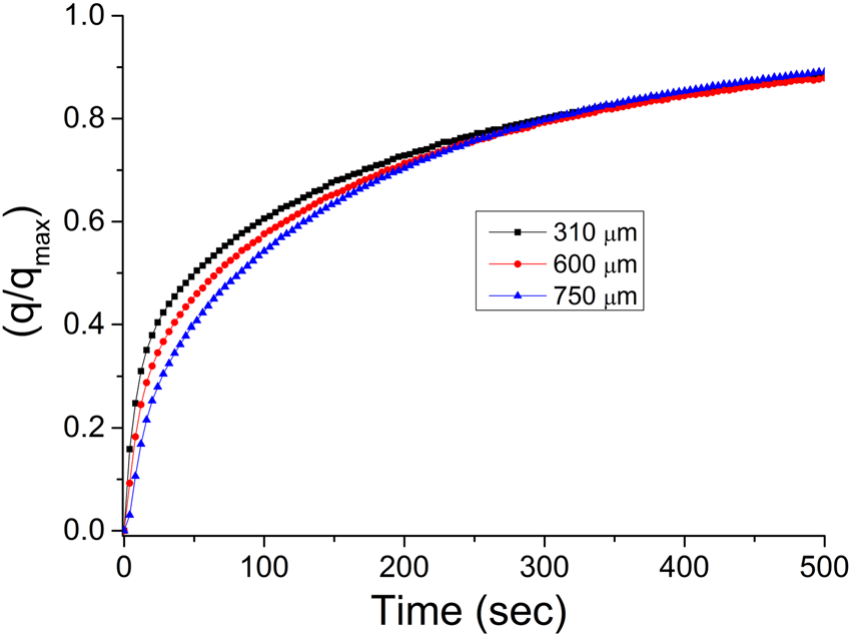
CO_2_ adsorption kinetics of laminates of varying thicknesses. The q/q_max_ gives the quantity of CO_2_ adsorbed at particular time (q) over the maximum CO_2_ adsorption capacity (q_max_) at saturation, after 3600 seconds in this study.

Figure 4 shows the CO_2_ uptake kinetics of NaX laminates of varying thicknesses. It is important to note that CO_2_ adsorption kinetics on laminates are compared in the second adsorption cycle. The structured zeolite laminates to capture CO_2_ are potentially suitable for pressure swing adsorption (PSA) and vacuum swing adsorption (VSA) processes, where the adsorbent is regenerated either by lowering the pressure or applying vacuum^5, 30^. Therefore, the contributions of chemisorbed CO_2_ observed on zeolites in first adsorption cycle^3, 31, 32^ are not relevant for estimates of the adsorption kinetics of a PSA/VSA process, where physisorption of CO_2_ will dominate. The crystallinity, BET surface area and CO_2_ adsorption capacity of the structured NaX laminates is similar to the NaX powder, which suggest that the durability of the laminates should be high and could sustain PSA/VSA cycles. The CO_2_ adsorption kinetics on NaX laminates in Fig. 4 shows that laminates with thickness of 310 micrometers has faster CO_2_ adsorption compared to the thicker laminates during the first 30–60 seconds. We find that the laminate of 310 μm thickness saturates to 40% of its maximum uptake capacity (q/q_max_ = 0.4) in 24 seconds compared to 36 seconds for the laminate with a thickness of 600 μm and 52 seconds for the laminate with a thickness of 750 μm. The faster CO_2_ uptake of thinner NaX laminate suggests that the CO_2_ uptake of structured laminates is macroporous diffusion controlled. However, it can be seen in Fig. 4 that the CO_2_ adsorption kinetics at longer times does not show significant difference on the thicknesses of the laminates suggesting that the diffusion mechanism becomes complex and other factors such as heat of adsorption, thermal conductivity, heat capacity of materials and heat transfer properties play role in controlling the long-time adsorption kinetics^32, 33^. The kinetic data suggests that it is of importance to design the structured laminate-based devices to effectively transfer the heat of adsorption from laminates for rapid CO_2_ adsorption kinetics.

## Conclusions

Pulsed current processing was used to produce binderless zeolite NaX laminates, 310 to 750 μm in thickness within 5% (15–20 μm) dimensional tolerances, for CO_2_ capture. NaX laminates demonstrated biaxial tensile strength in excess of 2 MPa. The adsorption capacity of CO_2_ was high 5.0 mmol/g, while the uptake of CH_4_ and N_2_ was relatively small; 0.4 mmol/g and 0.7 mmol/g, respectively. Ideal adsorption solution theory was used to estimate binary CO_2_-over-N_2_ selectivity (290 at 100 kPa at 25 °C) in 15 mol% CO_2_–85 mol% N_2_ gas mixture and CO_2_-over-CH_4_ selectivity (96 at 100 kPa at 25 °C) in 50 mol% CO_2_–50 mol% CH_4_ gas mixture. The uptake of CO_2_ of the NaX laminates is fast and a laminate with a thickness of 310 μm reach 40% of maximum uptake in only 24 seconds. The adsorption kinetics slows down with increasing laminate thickness but laminates with a thickness of 750 μm reach 40% of maximum uptake in less than one minute. The results suggested that NaX laminates hold great potential for selective separation of CO_2_ from N_2_ and CH_4_ in rapid swing adsorption processes.

## Experimental

### Materials

Zeolite NaX powder (Sigma-Aldrich GmbH, Germany) of 1–2 μm particle size was used as received.

### Processing

Zeolite NaX laminates were produced in graphite dies of 52 mm in diameter by pulsed current processing (PCP). Spark plasma sintering (SPS) equipment (Dr. Sinter 2050, Sumitomo Coal Mining Co., Ltd., Japan) was used. The mass of NaX powder was used to optimize laminates thicknesses from 310 μm to 750 μm. The graphite dies containing NaX powder were prepressed at 50 MPa and then placed between SPS electrodes with a graphite spacer assembly. NaX powder assemblies were heated to target temperatures between 500 and 800 °C and held for 3 minutes. The heating rate adopted was 100 °C min^−1^. 20 MPa compressive pressure was applied throughout the heating and holding time. The laminates were ejected from graphite dies after pressureless cooling in SPS machine down to 100 °C.

### Characterization

Field emission gun scanning electron microscope (FEG-SEM), JSM-7000 F (JEOL, Tokyo, Japan) was used to study the microstructure of NaX lamintes. Low accelerating voltage of 5 kV was used to avoid charging up of the surfaces under observation. A PANalytical X’Pert PRO powder diffractometer (PANalytical, Almelo, Netherlands) (Cu*K*_*α1*_ radiation λ = 1.540598 Å) was used to study the crystal structure of as-received powders and PCP processed laminates. The diffractometer was operated at at 45 kV and 40 mA and X-ray diffraction data was collected between 2θ = 5.0–60.0°. The biaxial tensile strength of the laminates was determined following ASTM F394 standard on a Zwick Z050 (Zwick GmBH Co & KG, Ulm, Germany). The biaxial tensile strength measurements were repeated for at least 5 laminates. Auto Pore III 9410 (Micromeritics, Norcross GA, USA) mercury intrusion porosimeter (MIP) was used to study the macropore volumes and pore size distributions in NaX laminates.

### BET surface area, CO_2_ and N_2_ adsorption and CO_2_ adsorption kinetics

ASAP2020 surface area analyzer (Micromeritics, Norcross GA, USA) was used to perform nitrogen adsorption-desorption measurements at −196 °C. Prior to measurements, the NaX powder and laminates were outgassed at 300 °C for 10 hours under near vacuum conditions. The nitrogen uptake at −196 °C in 0.05–0.15 *p/p*_*o*_ relative pressure range was used to determine the Brunauer-Emmet-Teller (BET) surface area of NaX powder and laminates. The same device, ASAP2020, was used for CO_2_, CH_4_ and N_2_ adsorption measurements at 20 and 25 °C from 0 to 101 kPa pressure. Prior to CO_2_, CH_4_ and N_2_ adsorption measurements, the zeolite NaX powders and laminates were outgassed at 300 °C for 10 h under near vacuum conditions. A thermogravimetric analyzer (TGA) (Setaram Instruments, Caluire, France) was used to study the CO_2_ adsorption kinetics on zeolite NaX laminates following the experimental conditions described elsewhere^18^.

### Adsorption models and Ideal adsorbed solution theory (IAST)

Langmuir isotherm model with two parameters was used to extract the parameters from CO_2_, CH_4_ and N_2_ adsorption isotherms. Three adsorption isotherms were used to extract parameters. These parameters were used as input for IAS theory estimation of binary adsorption selectivity $({\alpha }_{C{O}_{2}/{N}_{2}},{\alpha }_{C{O}_{2}/C{H}_{4}})$ following our previous work^18^.

## Electronic supplementary material

Supplementary Information
