# Supplementary material for: Thin zeolite laminates for rapid and energy-efficient carbon capture
Source: Sci Rep. 2017 Sep 8;7:10988. doi: 10.1038/s41598-017-10518-4 (PMC5591299; doi:10.1038/s41598-017-10518-4)
Supplement: Supplementary file 1 — Supplementary Information [file 41598_2017_10518_MOESM1_ESM.doc]

**Supplementary information**

**Thin zeolite laminates for rapid and energy-efficient carbon capture**

Farid Akhtar^1,2,*^, Steven Ogunwumi^3^, Lennart Bergström^2^

^1^ Division of Materials Science, Luleå University of Technology, 97187 Luleå, Sweden

^2^Department of Materials and Environmental Chemistry, Stockholm University, Stockholm 10691, Sweden

^3^Crystalline Materials Research, Corning Incorporated, USA

**S1: X-ray diffraction:**

X-ray diffraction (XRD) data of NaX powder and NaX laminate of 310 μm thickness produced by pulsed current processed (PCP) at 510 ◦C shows that the crystallinity of NaX is preserved after the thermal treatment.


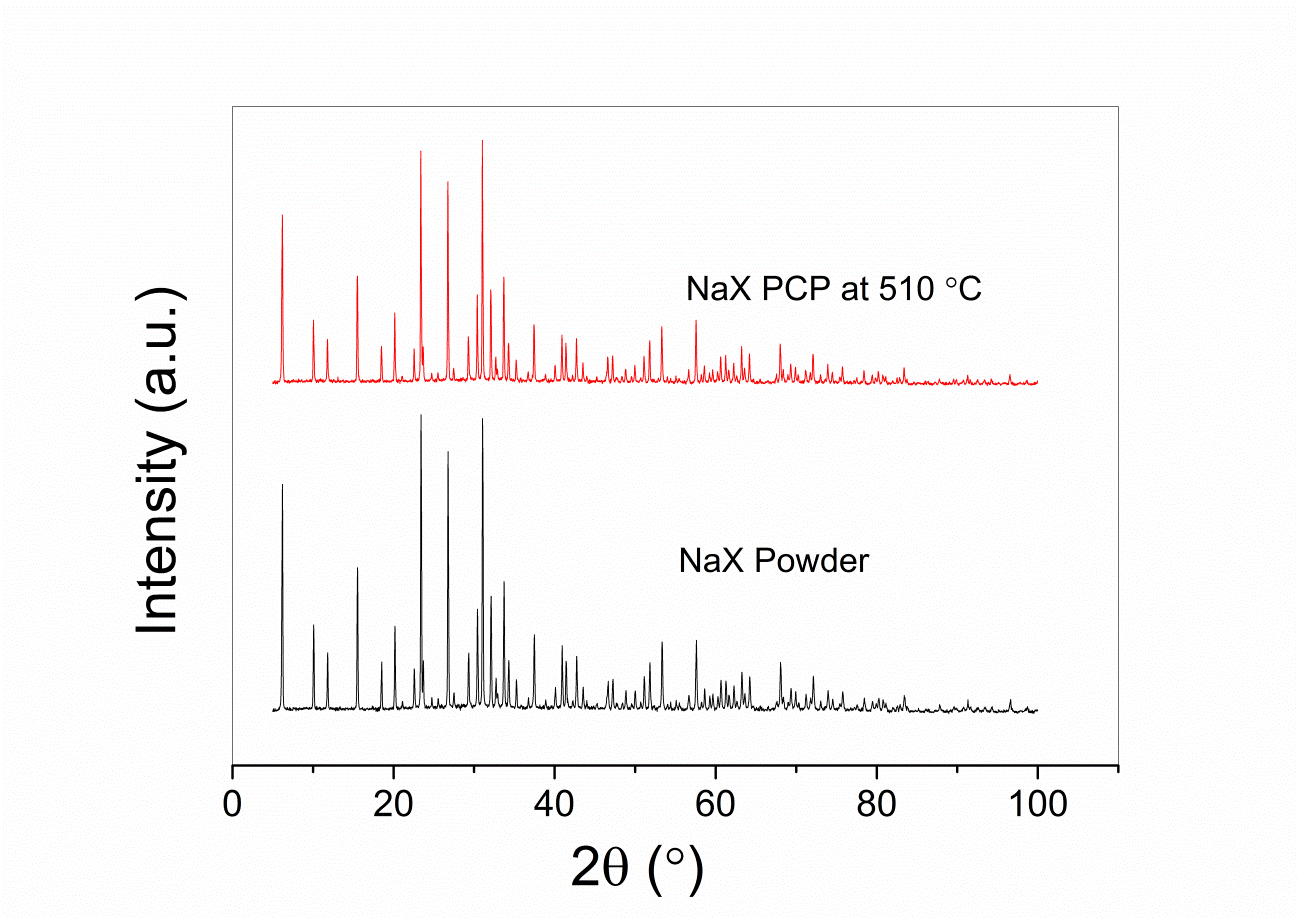


Figure S1: XRD pattern of NaX powder and NaX laminate (310 μm) PCP at 510 ◦C.

**S2: Adsorptive properties NaX powder:**

Table S1: The data of CO_2_, CH_4_, N_2_ adsorption and binary selectivity of NaX powder for the purpose of making a comparison with NaX laminates.

| Adsorbate | [a] q_m_ (mmol/g) | [a] b (1/kPa) | K_H_ (q_m_ x b) | [b] Binary CO_2_/N_2_ Selectivity | [b] Binary CO_2_/CH_4_ Selectivity |
| --- | --- | --- | --- | --- | --- |
| CO_2_ | 5.72 | 0.17 | 0.97240 | 262 | 79 |
| CH_4_ | 6.94 | 0.00138 | 0.00957 | --- | --- |
| N_2_ | 3.66 | 0.0016 | 0.00585 | --- | --- |

[a] Acquired from CO_2_, CH_4_ and N_2_ adsorption isotherm, respectively, at 293 K by implementing Langmuir model to data. [b] Calculated IAST at 100 kPa in 15 mol % CO_2_ and 85 mol % N_2_ and 50 mol % CO_2_ and 50 mol % CH_4_ binary mixtures at 100 kPa and 20 °C.
